# Flood risk in past and future: A case study for the Pawtuxet River's record‐breaking March 2010 flood event

**DOI:** 10.1111/jfr3.12655

**Published:** 2020-08-13

**Authors:** Soroush Kouhi, M. Reza Hashemi, Rozita Kian, Malcolm Spaulding, Matthew Lewis, Isaac Ginis

**Affiliations:** ^1^ Department of Ocean Engineering University of Rhode Island South Kingstown Rhode Island USA; ^2^ Graduate School of Oceanography University of Rhode Island South Kingstown Rhode Island USA; ^3^ School of Ocean Sciences Bangor University Bangor UK

**Keywords:** climate change, flood risk, HEC‐RAS, hurricane, river flooding

## Abstract

In March 2010, a sequence of three major rainfall events in New England (United States) led to a record‐breaking flooding event in the Pawtuxet River Watershed with a peak flow discharge of about 500‐year return period. After development of hydrological and hydraulic models, a number of factors that played important roles in the impact of this flooding and other extreme events including river structures (reservoirs, historical textile mill dams, and bridges) were investigated. These factors are currently omitted within risk assessments tools such as flood insurance rate maps. Some management strategies that should be considered for future flood risk mitigation were modeled and discussed. Furthermore, to better understand possible future risks in a warmer climate, another extreme flood event was simulated. The synthetic/hypothetical storm (Hurricane Rhody with two landfalls) was created based on the characteristics of the historical hurricanes that severely impacted this region in the past. It was shown that while the first landfall of this hurricane did not lead to significant flood risk, the second landfall could generate more rain and flooding equivalent to a 500‐year event. Results and the methodology of this study can be used to better understand and assess future flood risk in similar watersheds.

## 1 INTRODUCTION

River flooding is a major cause of catastrophic loss in the United States and around the world. Table 1 shows catastrophic loss by cause for about two decades in the United States (1996–2016^1^). Losses of 91.9% were caused by weather‐related events including tornadoes, hurricanes, and winter storms in which flooding has a significant contribution. Previous studies indicated that the risk of riverine flooding is increasing due to climate change (Booij, 2005), sea level rise (Le, Nguyen, Wolanski, Tran, & Haruyama, 2007) (for the rivers draining to the open seas and the ocean), and growth in populations/urbanization (Campbell et al., 2018; Suriya & Mudgal, 2012) in flood zones.

**TABLE 1:** Insured catastrophe losses by cause during 1997–2016 (adjusted for inflation)

Cause of loss	Loss (billion $)	Loss %
Tornadoes	168.1	39.9
Hurricanes and tropical storms	161.1	38.2
Other wind/hail/flood	29.7	7.1
Winter storms	28.2	6.7
Terrorism	25.0	5.9
Fires	8.4	2.0
Other	0.8	0.2
Total	421.2	100.0

In the United States, Federal Emergency Management Agency (FEMA, https://www.fema.gov/) generates Flood Insurance Rate Maps (FIRMs) for the assessment of flood risk in the flood zone. FIRMs provide a general guidance to estimate the river and coastal storm risk to structures/infrastructure in the flood zone corresponding to various exceeding probabilities or return periods (500 and 100 years). Nevertheless, several factors that can potentially affect the flood risk are not provided in those maps. For instance, management of river structures such as reservoirs and small dams can affect flood risk (e.g., dam removal/failures, how water elevation in a reservoir is managed during a flood event). Over 75,000 dams, including many small dams constructed during industrial revolution in the 18th century for textile industry, are built on rivers in the United States, while less than a 1000 of them have been removed (Shuman, 1995). Additionally, wood debris is abundantly found along the rivers and can enhance the flood risk upstream of bridges (Abbe & Montgomery, 2003). Trends in average and extreme precipitation due to climate change can affect the flood risk. It is not clear how these factors can change the estimated flood risk (e.g., 500 and 100 years) which are used in FEMA FIRMs.

In the Eastern United States, in addition to winter and spring storms, wet hurricanes can lead to major inland flooding. Hurricane Harvey (Category 4) is a recent example that led to more than a 100 cm of rainfall in some areas and caused a massive damage (Zhang, Villarini, Vecchi, & Smith, 2018). Previous studies also suggest that tropical cyclone intensity will increase as the climate warms (Sobel et al., 2016). Furthermore, extreme precipitations generated by recent wet hurricanes have been linked to climate change (Trenberth, Cheng, Jacobs, Zhang, & Fasullo, 2018). Therefore, there is a possibility of extreme precipitation events due to wet hurricanes as another cause of flood risk.

**FIGURE 1:**
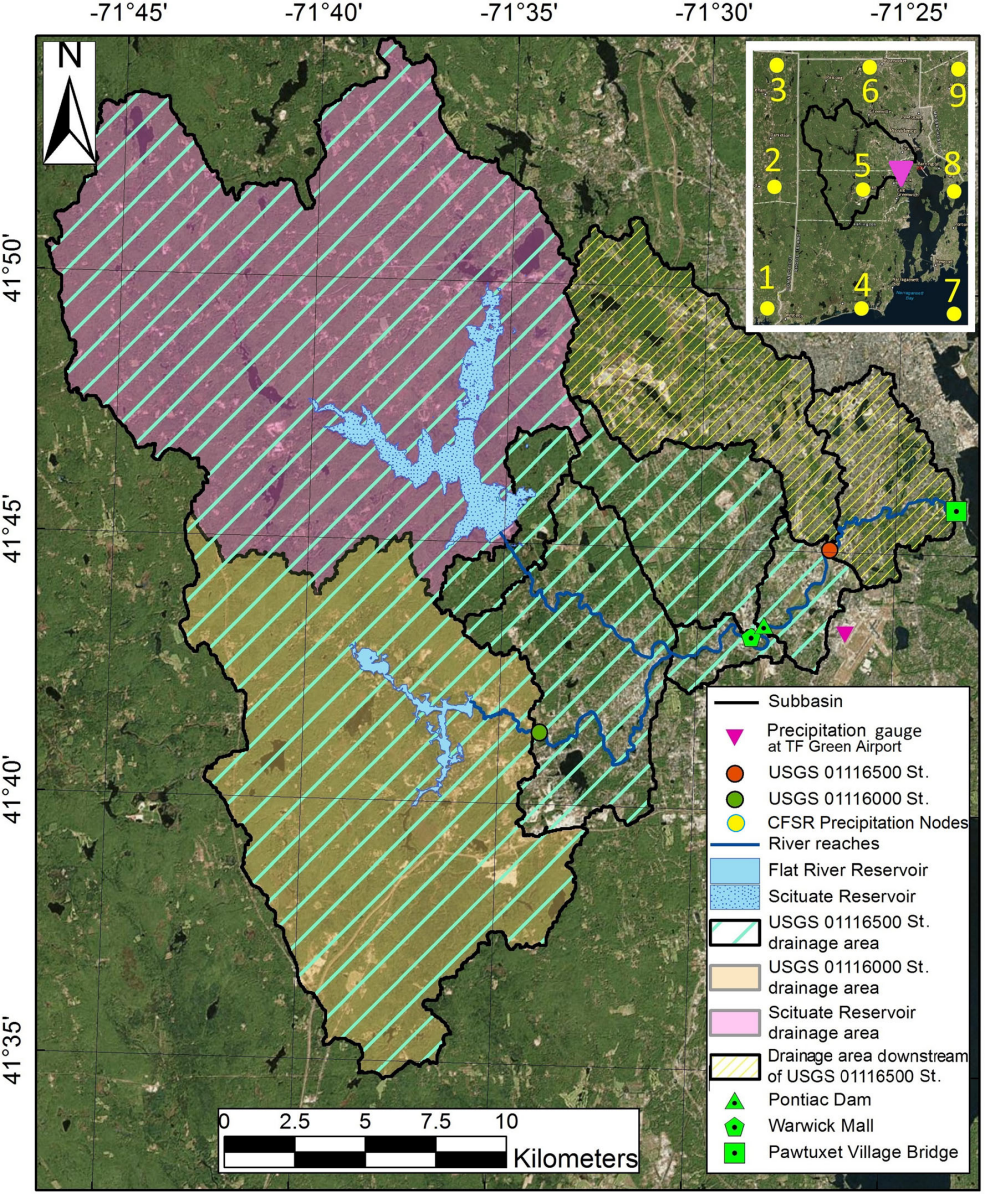
Map of the Pawtuxet River watershed. The watershed and subbasins borders are shown in black. The locations of the USGS stream gauges, meteorological station, reservoirs, and a few river structures are also shown. Locations of CFSR nodes to extract hindcast rainfall data are shown on the top right subfigure. CFSR, Climate Forecast System Reanalysis; USGS, United States Geological Survey

**FIGURE 2:**
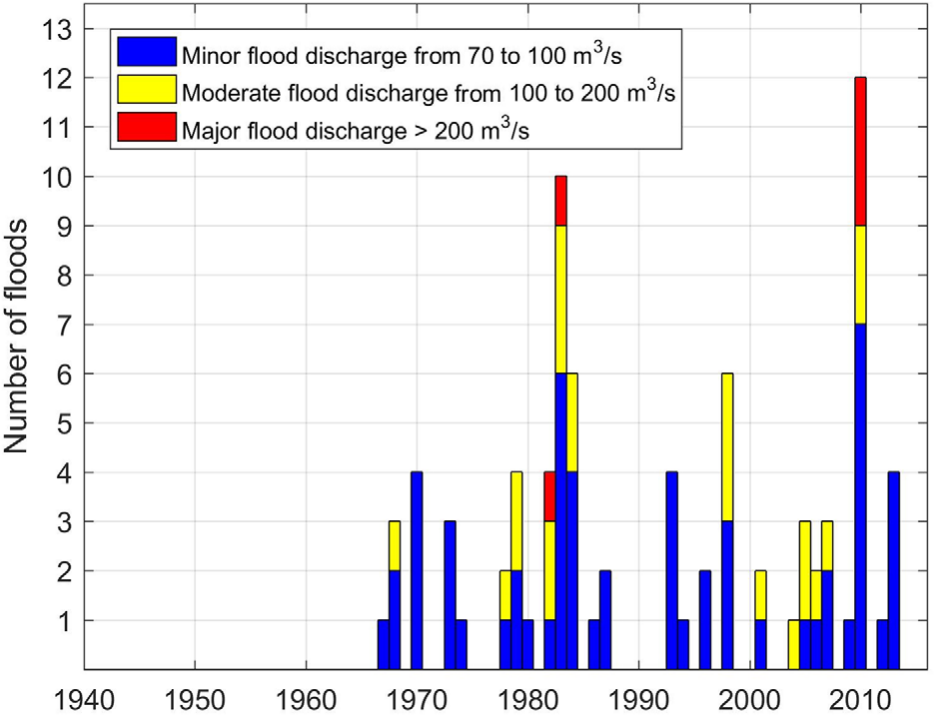
Historical flood frequency per year at the USGS 01116500 in Cranston from 1940 to 2017. The colour of the bars show the severity of the floods based on the flowrate. USGS, United States Geological Survey

Our case study, the Pawtuxet Watershed, is located on the western side of Narragansett Bay in Rhode Island (Figure 1), on the Northeast United States. The frequency of floods at the main United States Geological Survey (USGS) stream gauge on this river at Cranston is shown in Figure 2. As this figure shows, after the late 1960s, the frequency of flooding, the frequency of multiple floods over a given year, and severity of floods have been increasing during the past decades. In March 2010, there were three major rainfall and runoff events in New England, which led to the worst flooding event recorded in 200 years in the Pawtuxet River. Therefore, this event will be the focus of this study. Furthermore, Climate Solutions New England (CSNE^2^; initiated through the Sustainability Institute in the University of New Hampshire) has prepared an analysis for climate change that has occurred in the past decades and has forecasted these changes to 2,100 for the New England at the regional scales (Hayhoe et al., 2007). CSNE analysis indicates an increasing trend in the average precipitation based on the historical and forecasts data (i.e., 1960–2099) for low and high global greenhouse gas emission scenarios. For instance, the annual average precipitation in Rhode Island has increased 10–12% since 1960 and is expected to increase 27% during 1960–2099. Results from the cited study predict an increase in the number of extreme precipitation events, more frequent flooding, and more severe flooding for the time period 2020–2099. This is consistent with other research, which shows that extreme precipitation is increasing with temperature in moist and energy‐limited regions and decreasing in dry and moisture‐limited areas (Prein et al., 2017). Therefore, it is necessary to take into account these factors for risk assessments for future.

Here, we conducted a detailed assessment of the flood risk in past and future for a case study in Pawtuxet Watershed considering factors that are ignored in the existing flood risk maps. Some recommendations for flood risk management were discussed based on this assessment. Results and methods applied here can help other investigators and decision makers to better understand future flood risks and develop strategies to address them.

## 2 METHODOLOGY

### 2.1 Study area

The Pawtuxet River is located on the western side of Narragansett Bay in Rhode Island (Figure 1). It drains a watershed of 594 km^2^ into the Providence River in the upper Narragansett Bay. The Pawtuxet River watershed is the largest in Rhode Island and includes 12 Rhode Island communities. The river includes three branches where many structures such as dams and bridges exist on each of the branches. The North Branch with the length of 10.7 km starts at the Scituate Reservoir. The South Branch with the length of 14.7 km starts at Flat River Reservoir, and connects to the North Branch in Warwick. The Main Branch (18 km) begins at the confluence of the South and North Branches, and drains to the Providence River in upper Narragansett Bay. Two USGS stream gauges are located in the watershed: the USGS 01116500 on the Main Branch at Cranston and the USGS‐01116000 on the South Branch at Washington (see Figure 1). Table 2 provides more details about drainage areas in this watershed.

**TABLE 2:** Important drainage areas in the Pawtuxet River watershed (see Figure 1)

		Area (km^2^)	Percentage
	Total watershed	595	100
1	Drainage area of the Scituate reservoir on the north branch	235	40
2	Drainage area of the USGS 01116000 on the South Branch	162	27
3	Drainage area of the USGS 01116500 on the Main Branch (excluding items 1 and 2)	103	17
4	Rest of the watershed, downstream of the USGS 01116500	95	16

### 2.2 River structures and their role in flooding

In the aftermath of March 2010 event, the affected communities, stakeholders, and flood management authorities were looking for main causes of this catastrophe and what could have been done to reduce the impacts of this event, and future similar events. Several hydraulic structures and bridges are built in the river over the centuries. In particular, the impact of Scituate Reservoir and its dam (Gainer Memorial Dam) as the largest structure on the river has been an issue of interest. Furthermore, the majority of the historical diversion dams that were used for hydropower are still on the river and can affect the flood risk. Due to high vegetation of river banks, the impact of debris is another issue. The increased risk due to possible blockage of debris is not addressed in the FEMA maps. Details of these structures have been provided here. The impact of these structures on flooding will be investigated using numerical models.

#### 2.2.1 The Scituate Reservoir

Two relatively large reservoirs have been constructed in the watershed: the Scituate Reservoir (the largest) on the North Branch and the Flat River Reservoir on the South Branch. Here, for brevity, we only present the results related to the Scituate Reservoir. The Scituate Reservoir with a maximum storage of 148,000,000 m^2^ (corresponding to surface area of 13.7 km^2^) provides over 60% of the Rhode Island (RI)'s drinking water. The reservoir's main structures include an earth‐filled dam with a length of 975 m and a height of 30 m, and an ogee spillway with a crest elevation of 87.21 m (NAVD 88) and a crest length of 134.11 m. The drainage area of the reservoir is 235.5 km^2^ which is about 45% of the total watershed area of the Pawtuxet River.

#### 2.2.2 Historical dams

**FIGURE 3:**
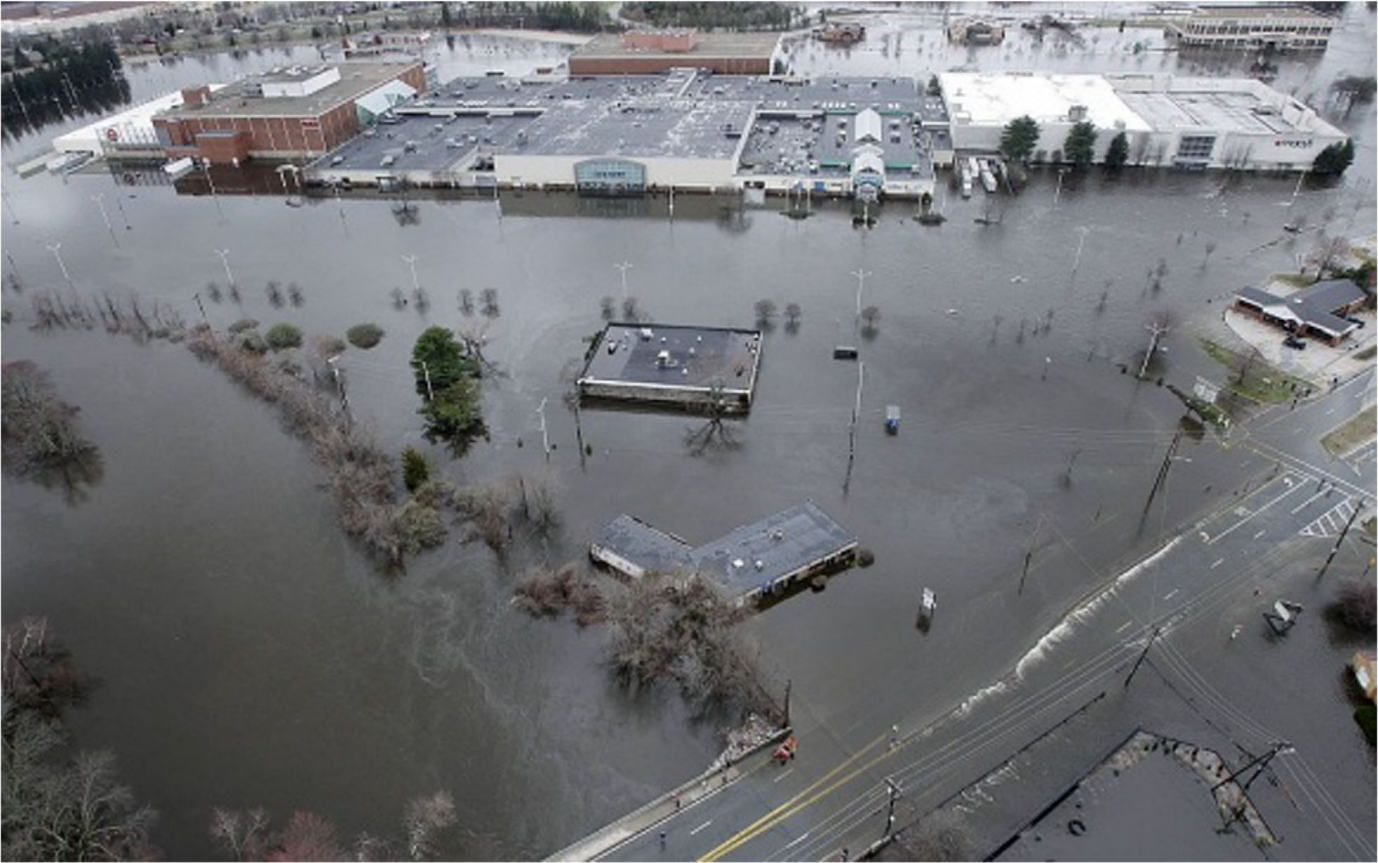
Flooding of Warwick Mall (in the Main Branch of Pawtuxet River) during March 2010 event (credit A. P.)

**FIGURE 4:**
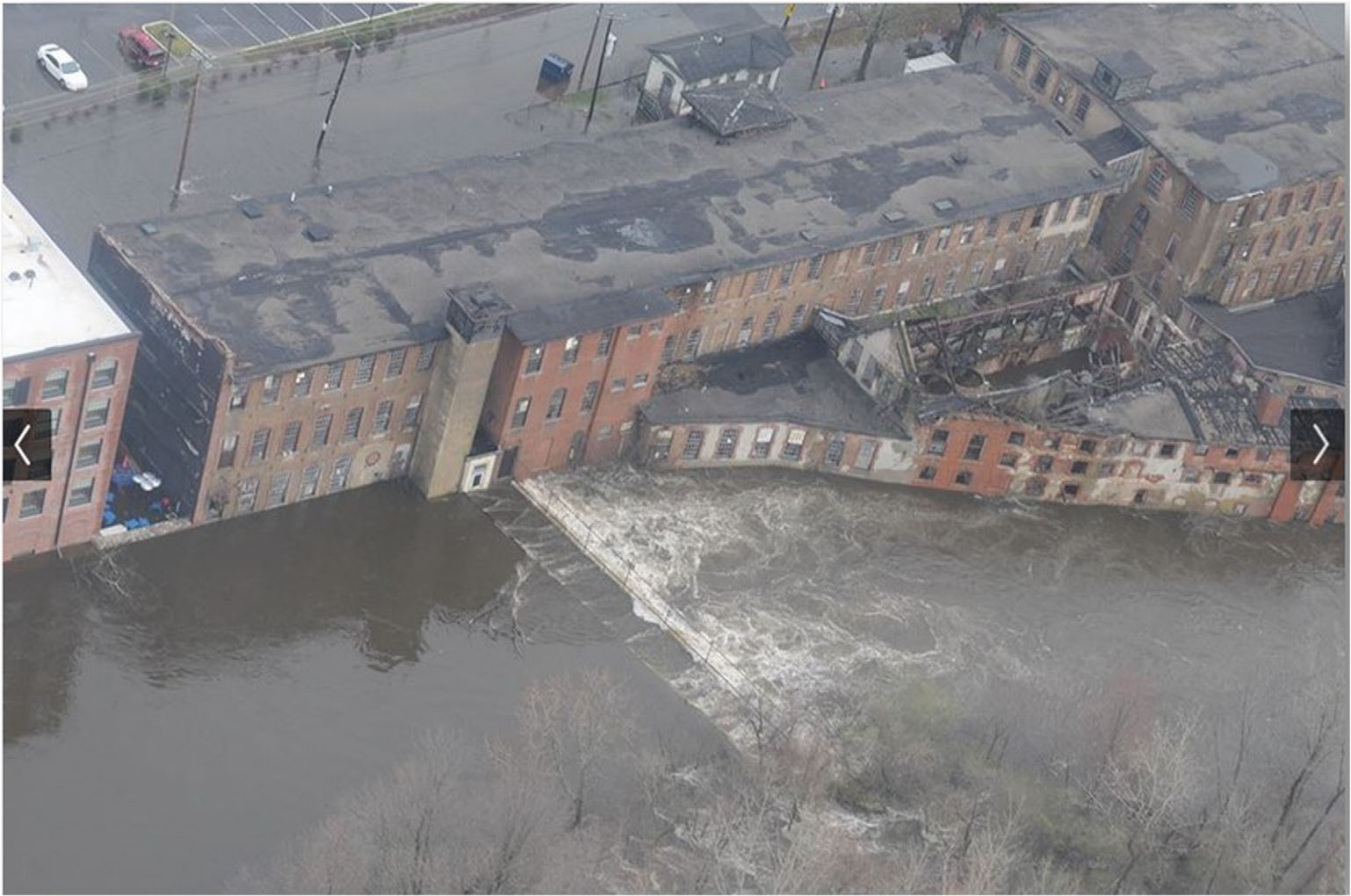
The aerial photo of the Pontiac Dam (in the Main Branch of the Pawtuxet River) during March 2010 event (Richardson, 2016)

Although textile mills were gradually decommissioned in New England and Rhode Island, the majority of the historical diversion dams that were used for hydropower are still in the river. There are 4 dams along the Main Branch, 8 dams along the North Branch, and 11 along the South Branch of the Pawtuxet River. Among those, the removal of the Pontiac Dam was discussed here because it is located in a highly commercial area, and just downstream of a large shopping center (Warwick Mall, Figure 3) which was flooded in the March 2010 event. Figure 4 shows aerial photos of Pontiac Dam during March 2010 flooding event. The location of Pontiac Dam and the Warwick Mall is shown in Figure 1. Pontiac Dam has a width of 30 m and a height of about 2.5 m.

#### 2.2.3 Debris accumulation at bridges

**FIGURE 5:**
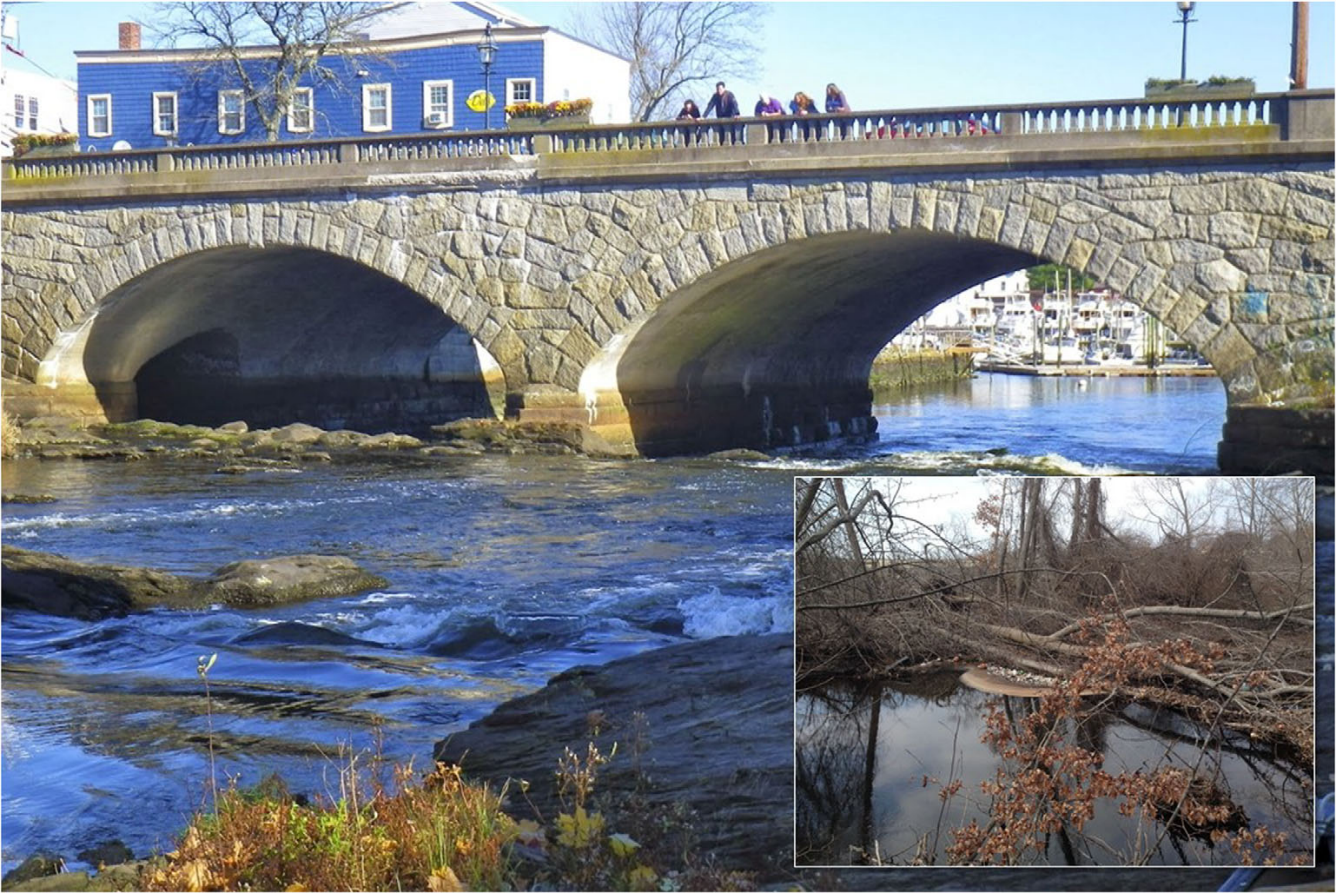
A snapshot of the Pawtuxet Village Bridge and some wood debris upstream of this bridge in the river channel that might be mobilized during a flooding event

Apart from dams, bridges can also affect the flood risk. In many rivers, debris such as broken trees can be frequently found in the floodplains and the watershed (Figure 5). Although debris can significantly increase the risk of flooding, its impacts on flooding are not considered in FEMA FIRMs. Additional obstruction due to debris results in decreased flow speed, reduced passage area, and increased inundation in areas upstream of a bridge. Along the Pawtuxet river and subbasins, there are 17 bridges in the Main Branch, 7 bridges in the North Branch, and 11 bridges in the South Branch.

### 2.3 Additional flood risk due to climate change and wet hurricanes

**FIGURE 6:**
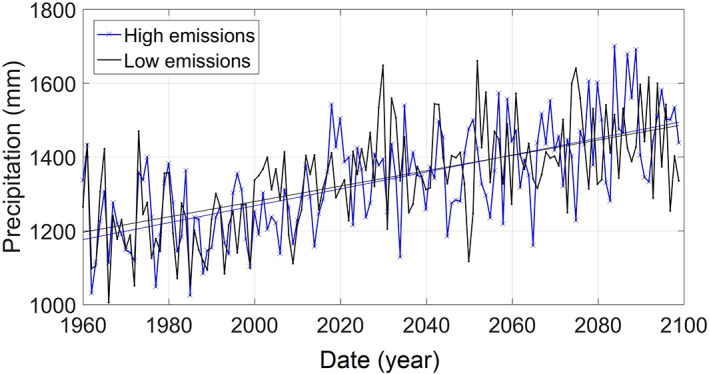
Rhode Island annual precipitation from 1960, and projected to 2099. Two projections are shown corresponding to low and high emissions scenarios (Hayhoe et al., 2007). The straight lines show linear trendlines for each scenario

Referring to Figure 6, the average annual precipitation in RI has increased 9.5% from 1960 to 2010. It is expected that the average annual precipitation will continue to increase up to 27% by 2,100 based on climate projections (Hayhoe et al., 2007). Some studies suggest that many regions in the United States may experience more intense extreme precipitation events up to 20%. The frequency of these extreme events is also expected to increase (e.g., Ragno et al., 2018) due to warming climate. (As mentioned before, Figure 2 shows that these changes are already happening in RI.) These studies recommend generating intensity–duration–frequency curves that include the trends due to changing climate (Cheng & AghaKouchak, 2014; Sarhadi & Soulis, 2017). Therefore, flood risk assessments should consider projected changes in precipitation due to climate change.

Seal level rise (SLR) is another consequence of climate change that can increase the flood risk. Apart from flow over very steep slopes (e.g., spillways, waterfalls, and steep mountain rivers), the flow regime in rivers is mainly subcritical. In a river/estuary with a subcritical flow regime, flooding extent is not only controlled by upstream inflow discharge, it also depends on water level at downstream (i.e., ocean water level). Therefore, tide and mean SLR can increase the risk of river flooding in areas near the ocean (Le et al., 2007). The Pawtuxet River empties into the Providence River in the upper Narragansett Bay. Therefore, water‐level variations in the Narragansett Bay will potentially impact the flooded areas in the Main Branch of the river. The projected SLR according to National Oceanic and Atmospheric Administration (NOAA), in the Extreme Scenario is about 3.5 *m* in 2100.

Previous research show a relationship between warming climate and the river flood risk. Extreme precipitation can occur as a result of various weather events. In the U.S. East Coast, in addition to winter storms, wet hurricanes and tropical storms can lead to extreme rainfall events (Zhang et al., 2018). In other regions such as the U.S. West Coast and United Kingdom, atmospheric rivers are one of the major causes of extreme flooding (e.g., Dettinger, Ralph, Das, Neiman, & Cayan, 2011). Atmospheric rivers have a long and narrow extratropical structure and can transport large amounts of moisture and lead to extreme precipitation. Some studies suggest up to 39% increase in the magnitude of atmospheric rivers in 2100 due to warming of climate (Nusbaumer & Noone, 2018). Recent studies also suggest that tropical cyclone intensity will increase as the climate warms (Sobel et al., 2016), and extreme precipitation of recent wet hurricanes has been linked to climate change (Trenberth et al., 2018). To assess the potential impact of a wet hurricane, a hypothetical wet hurricane that was created in a recent study is considered in this study (Stempel, Ginis, Ullman, Becker, & Witkop, 2018; Ullman et al., 2019). More details about this hurricane are provided in Section 3.

### 2.4 Data

The digital elevation model (DEM) with a resolution of 1 m was obtained from the light detection and ranging (LiDAR) ground elevations. Airborne LiDAR technology was applied to collect elevation data for the state of Rhode Island in detail from April 22, 2011 to May 6, 2011, which was part of the U.S. Northeast LiDAR Project.^3^ The Land Use/Land Cover data for the watershed were provided by the digital data set for the state of Rhode Island in Spring 2011. The classification scheme is similar to Anderson Level III modified coding in Rhode Island in 1988, 2003, and 2004 (Anderson, 1976). This data set is available through RI Geographic Information System (GIS) database.^4^ Soil type data were provided by the Web Soil Survey, produced by the National Cooperative Soil Survey. The USDA Natural Resources Conservation Service (NRCS) has provided soil maps and online data for more than 95% of the nation's counties.^5^ The NRCS classifies soils into four different Hydrologic Soil Groups: A, B, C, and D. Group A soils absorb water readily (e.g., sand). The subsequent groups have lower infiltration rates. Group D soils, like clay, do not allow significant water infiltration, causing more runoff. In general, the northern part of the watershed is mostly covered by soils classified as C, the western part by B, the eastern part by A, and the southern parts by the mixture of all the types. NOAA's National Centers for Environmental Information hourly precipitation data are available at the T.F. Green Airport in Warwick, RI. Flow discharge and water elevation data are provided at two USGS stream gauges 01116000 and 01116500 since 1940 (Figure 1).

**FIGURE 7:**
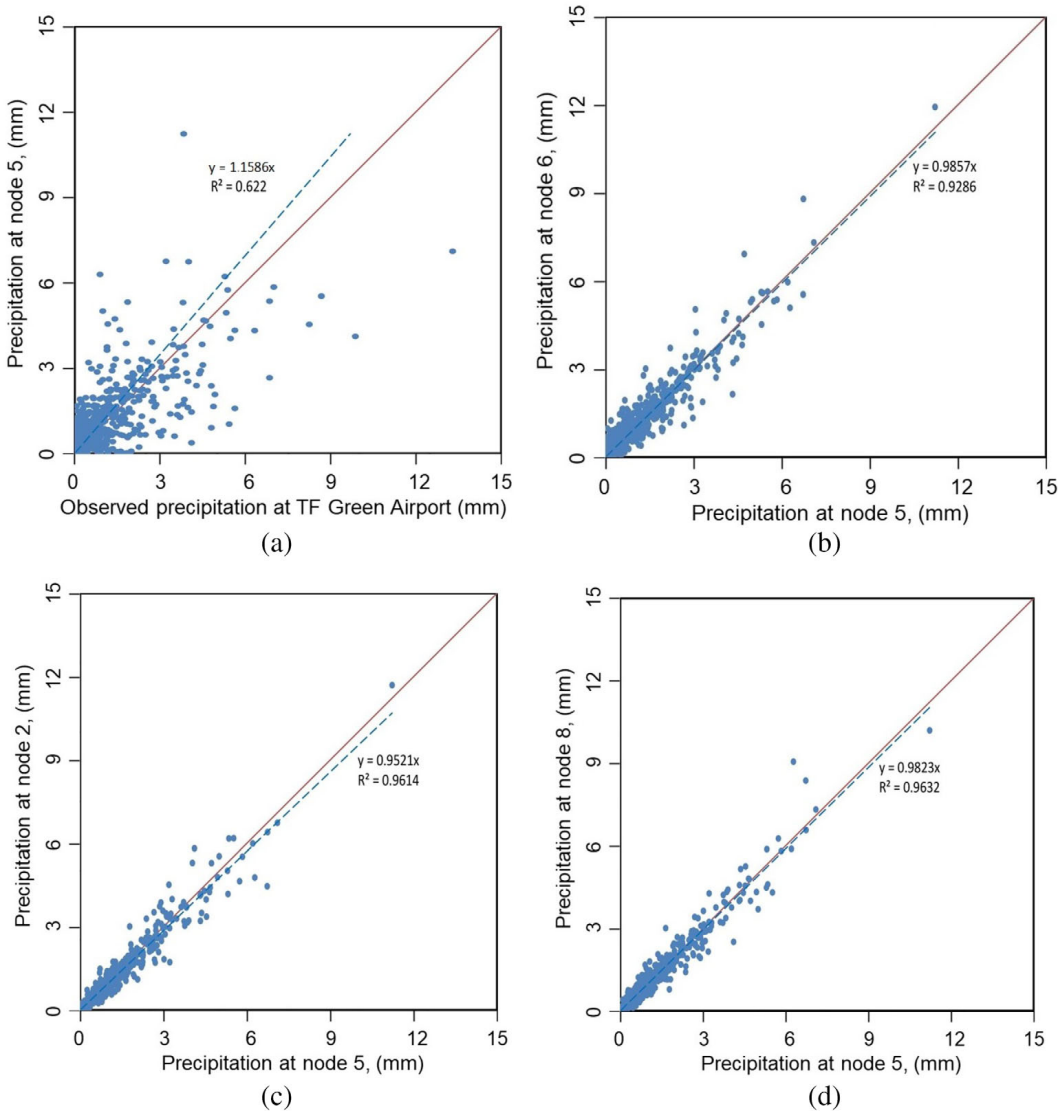
Spatial variability of daily precipitation in the area. Comparison of precipitation at various hindcast nodes as well as observed data are shown. See Figure 1 for locations of the nodes

The spatial variability of the rainfall can be significant in the watershed model. As mentioned, the T. F. Green Airport is the only precipitation station in the Pawtuxet River Watershed. In order to investigate the spatial variability of precipitation, we used available hindcast data from National Centers for Environmental Prediction Climate Forecast System Reanalysis (NCEP CFSR) database. Daily precipitation hindcast data can be extracted from this database. The locations of nine CFSR model nodes (closest to the watershed were chosen) and T. F. Green Airport Station are shown in Figure 1. Figure 7a compares the observed daily precipitation data of the T. F. Green Station with the closest CFSR model (Node 5) during 2008–2012 period. Figure 7b–d shows how precipitation varies among all nodes during 2008–2012 period. As these hindcast data show, the spatial distribution of the daily precipitation over the nodes is almost uniform and no significant gradient in the area that covers the watershed can be observed. This can be explained due to relatively small size of the watershed and also because the watershed is not mountainous (the maximum elevation difference is 75 m). A relatively good agreement of the watershed model and the observed data also shows the validity of this assumption.

### 2.5 Numerical modeling

**FIGURE 8:**
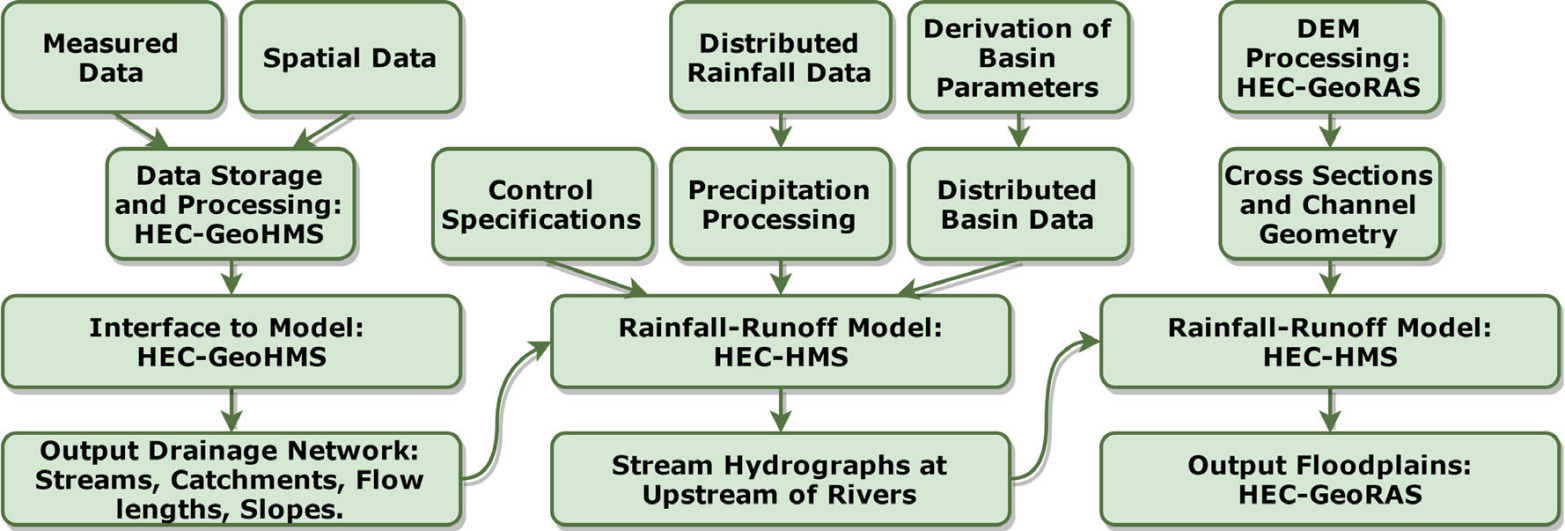
The flowchart of a distributed hydrologic and hydraulic modelling system for flood risk assessment (Knebl, Yang, Hutchison, & Maidment, 2005)

Hydrologic Engineering Center's Hydrologic Modeling System (HEC‐HMS) and River Analysis System (HEC‐RAS) were implemented to simulate runoff and river flooding, respectively. The detailed descriptions of these popular models can be found elsewhere (Brunner, 2001; Brunner, 2016; Feldman, 2000; Patel, Ramirez, Srivastava, Bray, & Han, 2017; Scharffenberg & Fleming, 2006). Figure 8 shows the steps applied in the modeling process. Spatial distributed data were preprocessed in ArcGIS and HEC‐GeoHMS to produce the drainage network, and build the rainfall‐runoff model in HEC‐HMS. The HEC‐HMS model requires distributed basin data (calculated in HEC‐GeoHMS), meteorological data, baseflow, and modeling control specifications (i.e., time interval and duration) to simulate and route the runoff as a result of a precipitation event. The time series of flow discharge calculated by HEC‐HMS were used as input/boundary condition in the river model (HEC‐RAS). Additionally, river cross sections (provided by USGS), channel geometry, river structures (geometry of bridges and dams along the river), and channel roughness are required by HEC‐RAS. Some of the geometric data were provided by USGS (G. Bent, personal communication, 16 November 2015), and some were extracted using the DEM and HEC‐GeoRAS. Along the river and sunbasins, there are 17 bridges and 4 dams in the Main Branch, 7 bridges and 7 dams including the Scituate Reservoir Dam in the North Branch, and 11 bridges and 11 dams including the Flat Reservoir Dam in the South Branch. Simulated water elevations by HEC‐RAS were then superimposed with the DEM to compute the flood maps in HEC‐GeoRAS and GIS environment.

HEC‐HMS has various options and methods that can be selected for runoff modeling. The methods used in the rainfall‐runoff calculations in HEC‐HMS were Soil Conservation Service (SCS) runoff Curve Number (CN) for surface runoff calculation, monthly constant method for the baseflow calculation, SCS unit hydrograph for subbasin flow routing, and lag time method for reach routing calculations. Selections are mostly based on the similar studies in the United States (Knebl et al., 2005; Williams, Kannan, Wang, Santhi, & Arnold, 2012). Other choices may also lead to similar results by proper calibration, but the most appropriate methods should be based on the physical characteristics of a watershed (Bhadra, Bandyopadhyay, Singh, & Raghuwanshi, 2010). The terrain preprocessing was carried out based on a 30 m resolution DEM in HEC‐GeoHMS. Terrain preprocessing includes stream definition, watershed delineation, and computation of subbasins properties. The watershed was divided into nine subbasins which included points of interest such as large reservoirs (Scituate and Flat River) and USGS stream gauges (validation stations).

**FIGURE 9:**
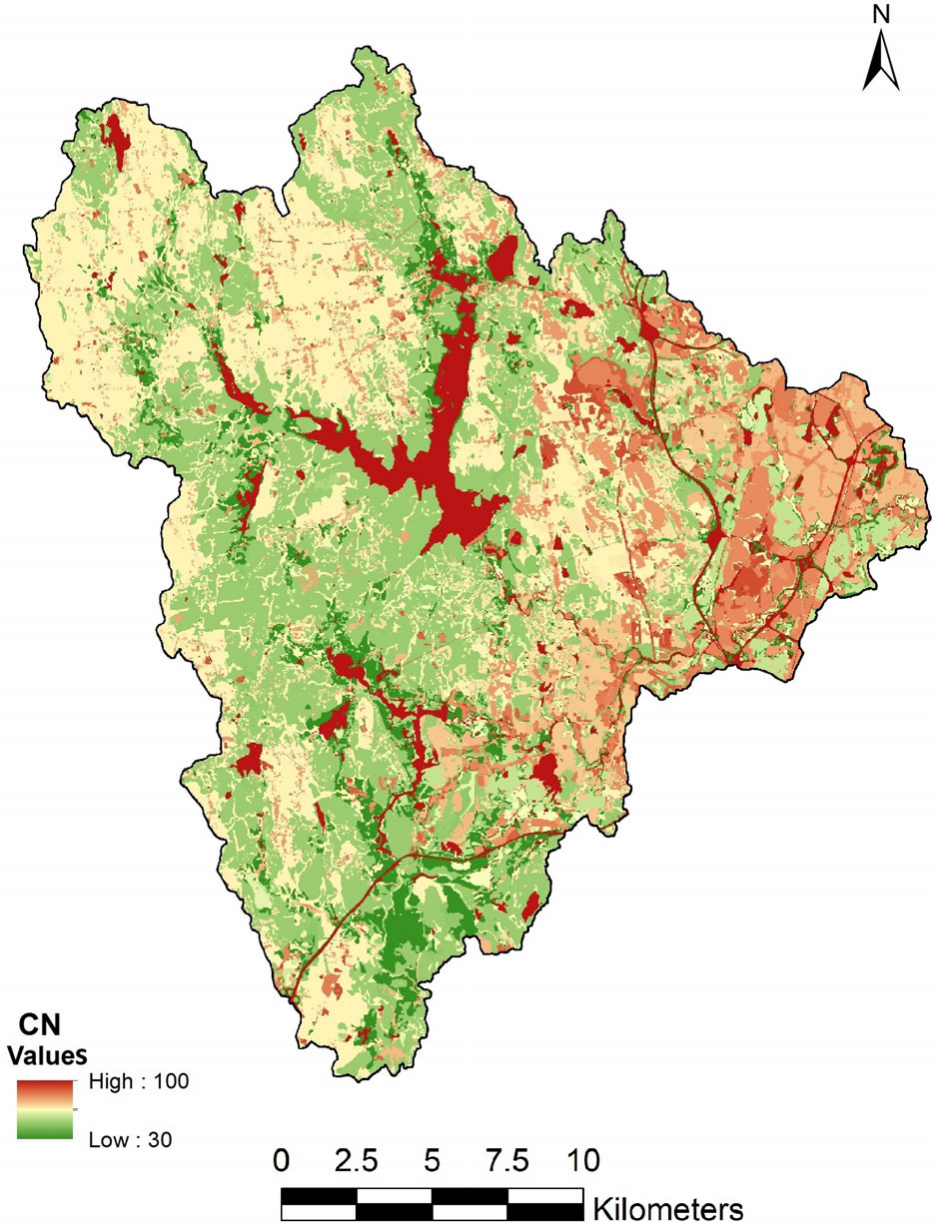
The CN map of the Pawtuxet River watershed. High CN values indicate reservoirs (water bodies) and urbanized areas (east region) that generate more runoff. CN, Curve Number

In the SCS method, the infiltration and runoff are predicted based on the empirical loss rate parameter (CN), which depends on the soil type, land use, and hydrologic condition. The direct runoff is estimated as (Feldman, 2000),

(1) $$P_{e}=\frac{{\left(P-0.2S\right)}^{2}}{P+0.8S},$$

where *P*_e_ is excess rainfall (direct runoff), *P* is precipitation, and *S* is potential maximum soil moisture which in SI units is evaluated as,

(2) $$S=\frac{25,400-254\mathrm{CN}}{\mathrm{CN}},$$

where 30 ≤ CN ≤ 100. In order to calculate CN, the soil type data and land cover data were combined in the GIS environment. A lookup table was used to evaluate the spatial variation of CN (Cronshey, 1986). The resulting CN map of the Pawtuxet watershed is shown in Figure 9. CN values are higher in the eastern (more urbanized) areas of the watershed which leads to more runoff. The estimated routing time lags varied from 100 to 600 minutes. The baseflow values for the subbasins varies monthly and was provided using USGS data.

HEC‐RAS was used both in the steady‐ and unsteady‐state modes. For steady‐state case, the peak discharge for each river reach was used to estimate maximum flood extent. The unsteady mode (which is computationally expensive and time consuming) was used as a comparison. HEC‐GeoRAS used the 1‐m resolution DEM for mapping the flood or complete missing geometric data (e.g., flood plains of some cross sections). In general, the Manning coefficients were set based on FEMA Flood Insurance Studies of the region (https://www.fema.gov/), and also using the USGS reference for roughness characteristics of natural channels (Barnes, 1967). The Manning coefficients was initially set to 0.04 for the main channel and increased to 0.08 for the river banks due to increase in vegetation cover. Further tuning were carried out to change these numbers at various cross sections to calibrate the HEC‐RAS model based on the observed high water marks as will be explained in the calibration section.

The flow hydrographs produced by HEC‐HMS were applied as the upstream boundary conditions. For the steady‐state cases, the peak discharge or the computed discharge for specific return periods (Zarriello, Ahearn, & Levin, 2012) were applied. The downstream boundary condition was the stage hydrograph that was extracted from NOAA database at a nearby station in Narraganset Bay: NOAA Providence Station 8454000.

#### 2.5.1 Calibration

**FIGURE 10:**
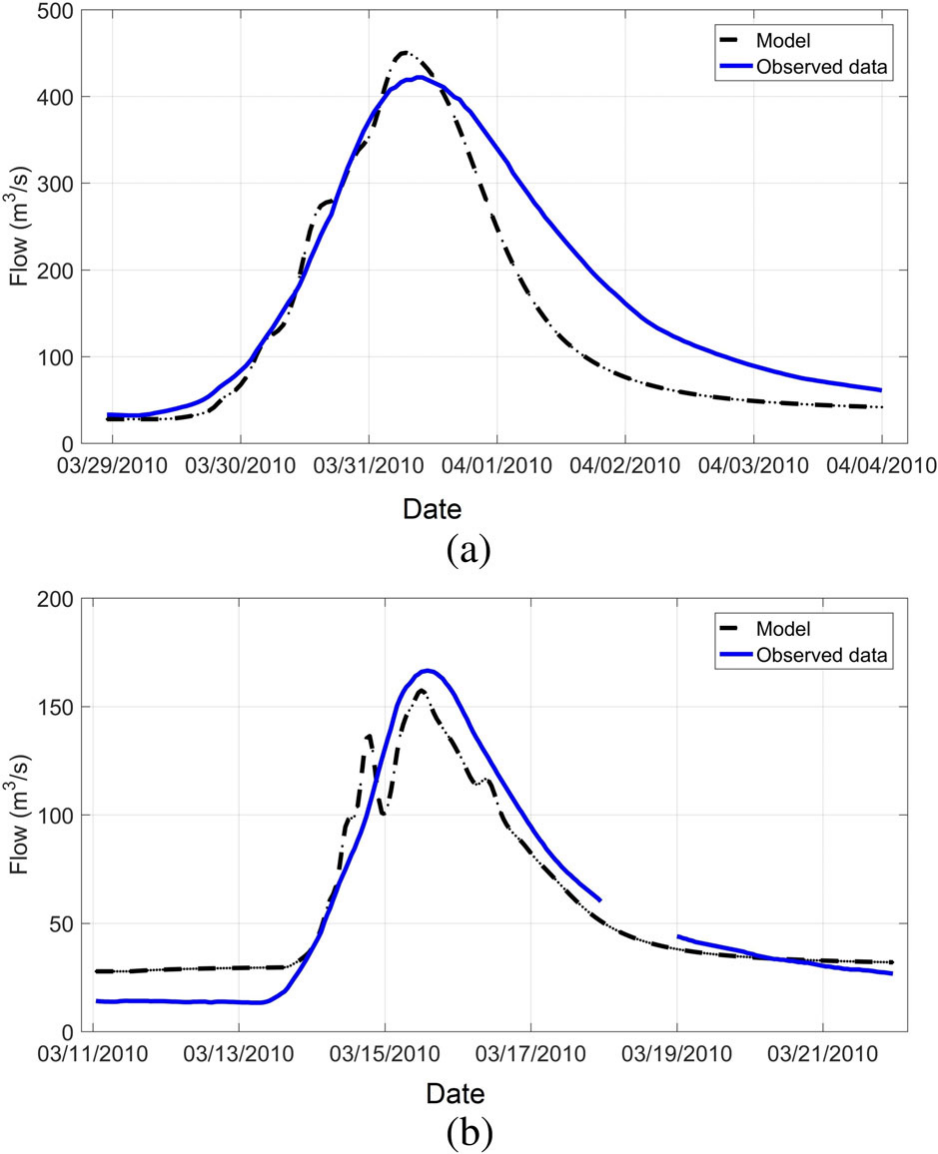
Comparison of hydrographs based on the observed data and calibrated HEC‐HMS model for (a) March 29 to April 4, 2010 (peak error of 6%), and (b) March 11 to March 21, 2010 (peak error of 6%) at USGS 01116500 in Cranston, RI. HEC‐HMS, Hydrologic Engineering Center's Hydrologic Modeling System; USGS, United States Geological Survey

Model calibration is a critical step to develop an accurate watershed model. For model calibration, a starting set of model parameters was selected, and runoff and peak flow discharge were calculated. The parameters were adjusted (automatically in HEC‐HMS) until the computed runoff best matched the observed data. To calibrate the watershed model, two events from 2010 in which hourly precipitation data were available from the T. F. Green Airport Station were selected. The first event was started at 11:00 p.m. on March 28, 2010 and continued until 12:00 a.m. on April 4, 2010: the record‐breaking flood event in this area; the second event started at 1:00 a.m. on March 11, 2010 and lasted until 11:00 p.m. on March 21, 2010. The depth of precipitations (rainfall) were 224 mm and 140 mm, for the first and second events, respectively. Volume of runoff and peak discharge were the two model output parameters in the calibration process. Parameters such as time lag and CN in a feasible range were adjusted automatically to calibrate the model. Based on the model results, the estimated CN values based on the land use and soil cover produced convincing runoff values. However, time lags of subbasins needed to be adjusted to minimize the discrepancies of observed and calculated peak flow discharge. During the calibration process, the Scituate Reservoir was considered to be at full capacity (i.e., at its maximum water level of 87.21 m NAVD88) which is consistent with the reservoir water level data provided by National Weather Service. Figure 10 compares the hydrographs of the observed and simulated discharges for the two selected events at the USGS station 01116500 in Cranston. Model errors are about 6% for the peak discharges at both events.

**FIGURE 11:**
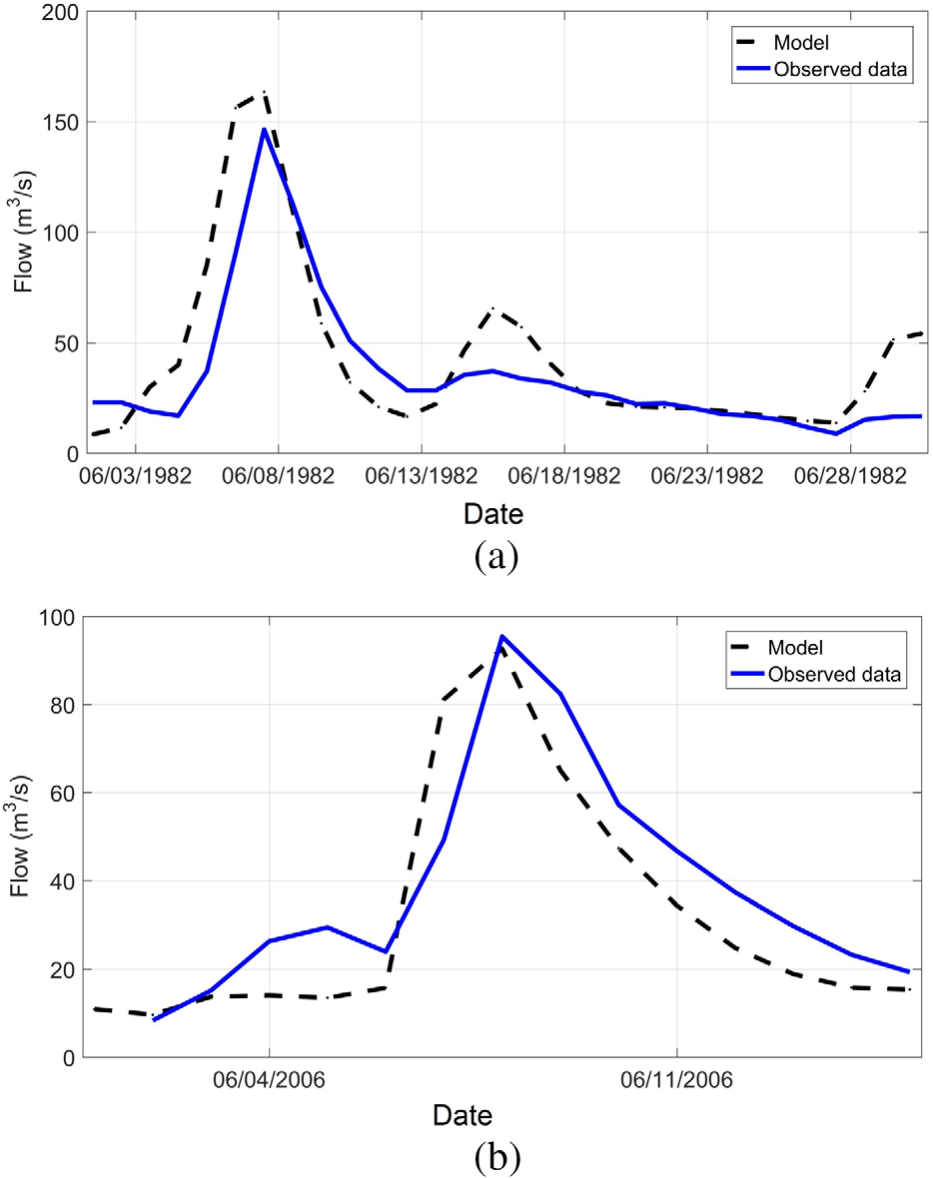
Comparison of the HEC‐HMS model results and the observed data for (a) June 1982 (peak error of 10%), and (b) June 2006 (peak error of 3%) at USGS 01116500 in Cranston, RI. HEC‐HMS, Hydrologic Engineering Center's Hydrologic Modeling System; USGS, United States Geological Survey

**FIGURE 12:**
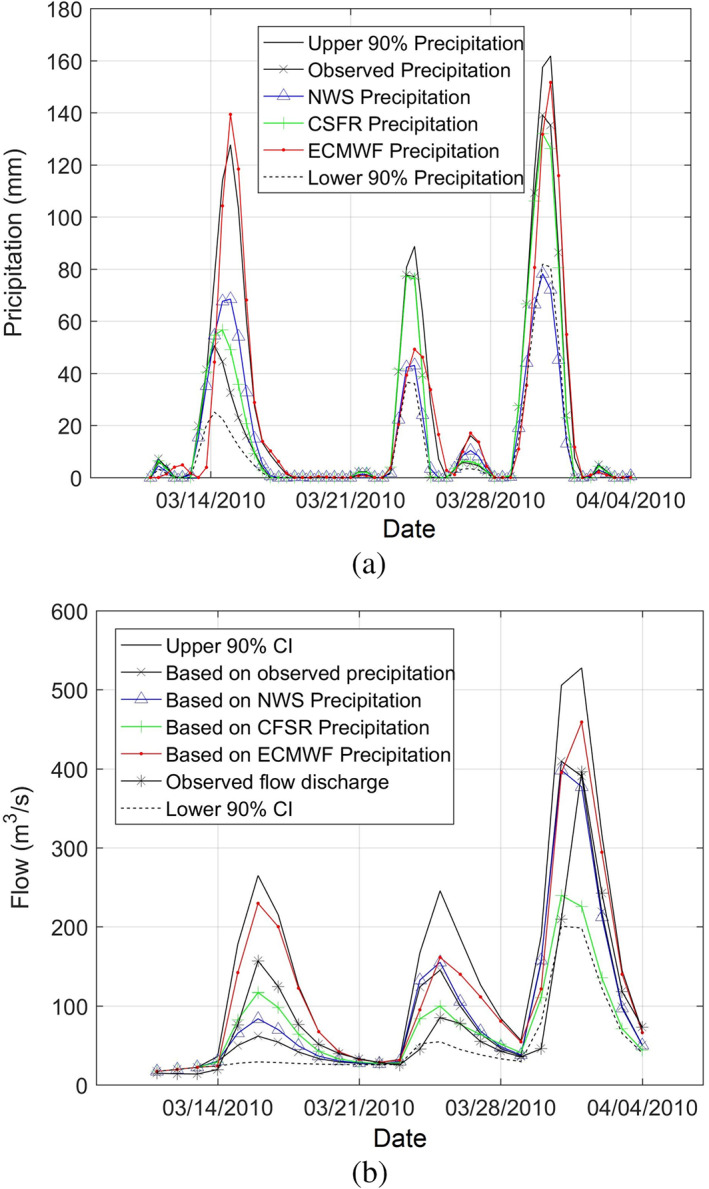
Effect of precipitation data on simulated flow discharge at USGS 01116500 in Cranston (a) Uncertainty in the several sources of the precipitation data: observed precipitation, NWS, ECMWF, CFSR, upper 90% limit, and lower 90% limit, and (b) Uncertainty in the HEC‐HMS flow discharges corresponding to several sources of precipitation data. CFSR, Climate Forecast System Reanalysis; ECMWF, European Centre for Medium‐Range Weather Forecast; HEC‐HMS, Hydrologic Engineering Center's Hydrologic Modeling System; NWS, National Weather Service; USGS, United States Geological Survey

The performance of the model was also assessed for two events which produced significant flow discharges (and we could find a continuous observed rainfall‐runoff record for them considering data gaps): June 1, 1982 to June 30, 1982 and another event from Jun 1, 2006 to Jun 15, 2006 using observed discharge data from the Cranston stream gauge (Figure 11). The model hydrographs for these two events compare relatively well in terms of number of peaks and peak discharge values. Apart from model parameters such as CN and lag time, an important source of uncertainty is precipitation data because there is only a single precipitation gauge near the watershed. Here, in addition to the precipitation data at T. F. Green Airport Station, three alternative precipitation data sets were examined: European Centre for Medium‐Range Weather Forecast (ECMWF), NCEP CFSR, and hindcast data provided by National Weather Service (NWS) (D. Vallee, personal communication, 2017). Figure 12(a) shows the precipitation time series for observed, ECMWF, CFSR, and NWS data sets as well as the upper 90% and lower 90% confidence intervals for the period that led to March 2010 event. Figure 12b shows the discharge time series corresponding to these time series. The calculated hydrograph based on the observed rainfall is in a good agreement with observed discharge data as expected from model calibration. However, there is a significant uncertainty in terms of precipitation data which lead to errors in estimation of discharge; this uncertainty cannot be addressed by adjusting the parameters of rainfall‐runoff model. This issue is particularly important while using the model for forecasting purposes when observed data are not available. Installing more precipitation gauges in the watershed (particularly upstream of Scituate reservoir) will reduce the uncertainty for both hindcast and forecast purposes.

**FIGURE 13:**
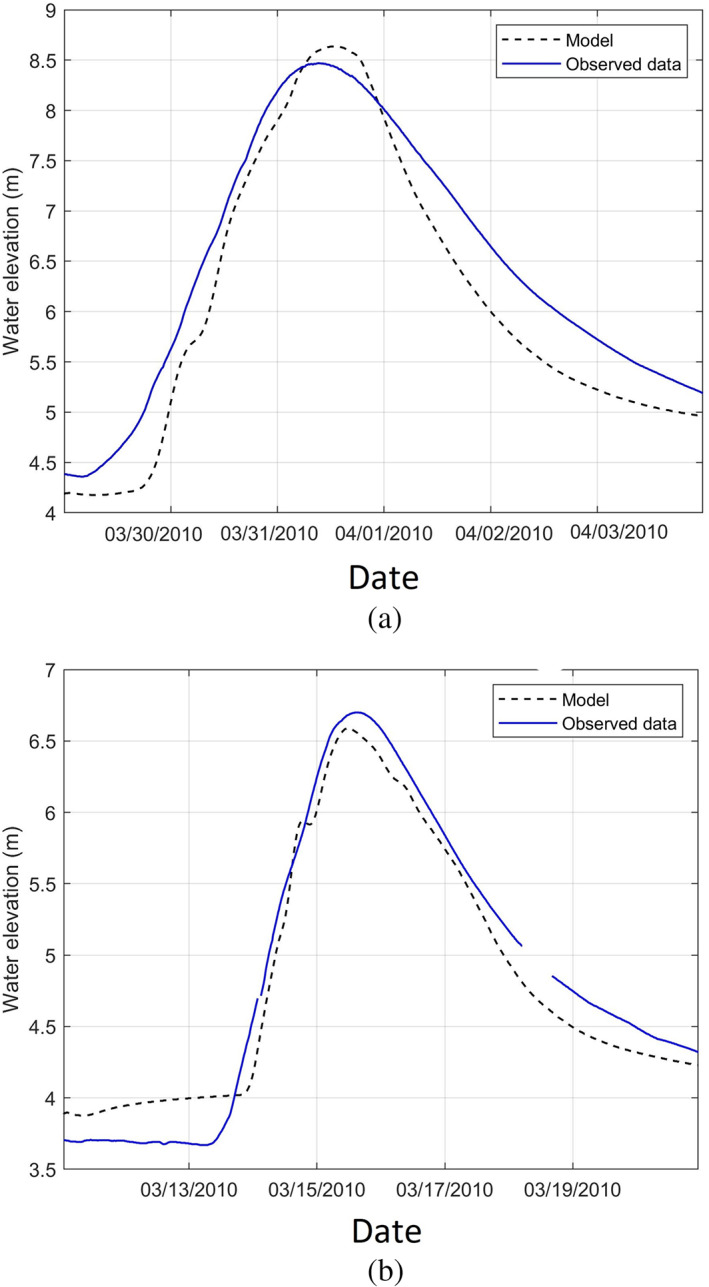
Comparison of modelled (HEC‐RAS) and observed stage hydrographs at USGS 01116500 in Cranston, RI; (a) March 29 to April 4, 2010 (2% error for peak elevation), and (b) March 11 to March 21, 2010 (1.8% error for peak elevation). Elevations are in NAVD 88. HEC‐HMS, Hydrologic Engineering Center's River Analysis System; USGS, United States Geological Survey

The HEC‐RAS model was calibrated for the same events as HEC‐HMS model: March 28, 2010 to April 4, 2010 and March 11, 2010 to March 21, 2010. Channel roughness (i.e., Manning coefficient) is a sensitive parameter which can be used to the calibrate a river hydrodynamic model. The Manning coefficient was adjusted for cross sections along the river. Several High Water Marks (HWM) obtained from a previous USGS study (Zarriello & Bent, 2011) were used to calibrate the model based on water elevations. Figure 13 compares the time series of water elevation (stage hydrographs) for the observed and model data at the USGS station 01116500 in Cranston. The results show a good agreement between HEC‐RAS model results and the observed data.

### 2.6 Overview of the model scenarios

After the development of the watershed and river models, several simulations were performed to assess the flood risk and how various factors can affect it. Table 3 summarizes the simulation scenarios. Based on Zarriello et al. (2012), flow discharges corresponding to the 50, 100, and 500 years were estimated as 196, 250, and 424 m^3^/s, respectively at the USGS Gauge 01116500 in Cranston.

**TABLE 3:** Overview of model runs

Category	Contributing factor	Model scenario
River structures	Scituate Reservoir	Full reservoir during 2010 flood event
		Partially filled reservoir during 2010 flood event
	Poniac Dam	50‐year event with and without the dam
		500‐year event with and without the dam
	Pawtuxet Village Bridge debris	50‐year event with and without debris
		500‐year event with and without debris
		100‐year event estimated before 2010
Climate change	Change in precipitation	100‐year event estimated after 2010
		100‐year upper 95% CI estimated after 2010
		100‐year event considering mean sea level
	Sea level rise (SLR)	100‐year event during high tide and surge
		100‐year event considering SLR downstream
	Extreme wet hurricane	Hurricane Rhody's synthetic rainfall

## 3 RESULTS AND DISCUSSION

### 3.1 Modeling the impact of river structures on flooding

**FIGURE 14:**
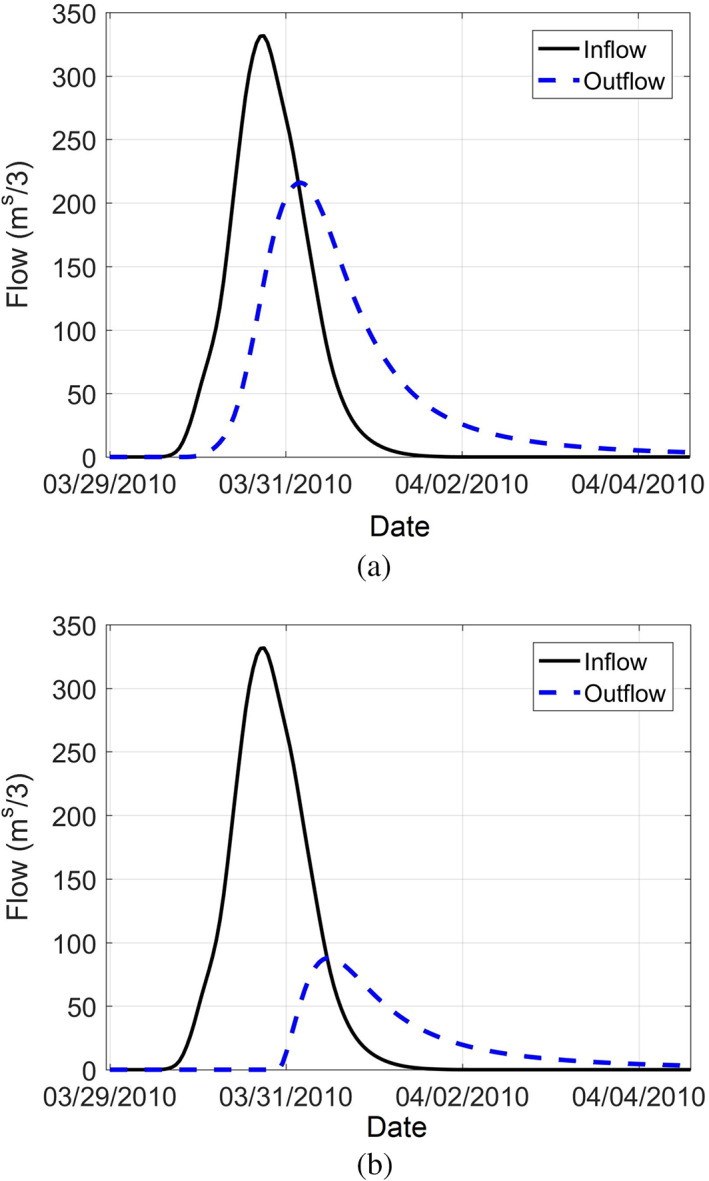
Inflow and outflow hydrographs at the Scituate Reservoir during March 2010 event; (a) assuming a full reservoir; (b) when the initial water level at the reservoir is assumed 1.2 m below the spillway crest

Using the watershed and river models, the impact of river structures (described in Section 2.2) on flooding and some recommendations for flood risk management are discussed here. Due to similarities of this case study and other watersheds in the northeast of the United States and elsewhere, these results will be of interest of flood management researchers and decision makers. Although Scituate reservoir was constructed and operated for water supply purposes, it has a significant role in flood risk. According to reservoir surface elevation data (D. Vallee, personal communication, 2017), during March 2010 event, the reservoir was at its full capacity. Using volume‐elevation curve and other detailed characteristics of the reservoir and spillway, the reservoir was modeled in HEC‐HMS. As an initial estimate, the total runoff of March 2010 event upstream of the reservoir was about 28 MCM. This volume of runoff could be captured in the reservoir with approximately 2 m of capacity (considering the reservoir's surface area of 13.8 km^2^). As an additional analysis, Figure 14 compares the simulated inflow and outflow at the reservoir for a full capacity (87.21 NAVD88), and 1.21 m below full capacity (86.00 NAVD88). As the figure shows, the peak outflow discharge will reduce around 60% (from 216 to 88 m^3^/s) if 1.21 m capacity is allocated to flood storage. This is equivalent of reducing the return period from 500 years (very extreme) to 10 years (moderate flood event).

Therefore, management of this reservoir has a crucial impact on flooding in this area. Since the original design of the reservoir was only based on water supply purpose, more study and initiatives are necessary to optimize the reservoir operations to minimize the flood risk and meet water supply demand (considering other constraints). Methods to add the capacity of the reservoir (e.g., gates‐controlled spillways instead of stop‐logs; Sordo‐Ward, Garrote, Bejarano, & Castillo, 2013) should be considered in this assessment. Furthermore, due to uncertainty in future extreme precipitation events as well as stress associated with a growing water demand, traditional reservoir management techniques that are based on historical data may not be that effective. More advanced reservoir management techniques (Ahmad, El‐Shafie, Razali, & Mohamad, 2014) that consider the uncertainty in flood risk and can adapt to new data should be employed (e.g., Georgakakos et al., 2012; Raje & Mujumdar, 2010; Uysal, Schwanenberg, Alvarado‐Montero, & Şensoy, 2018).

**FIGURE 15:**
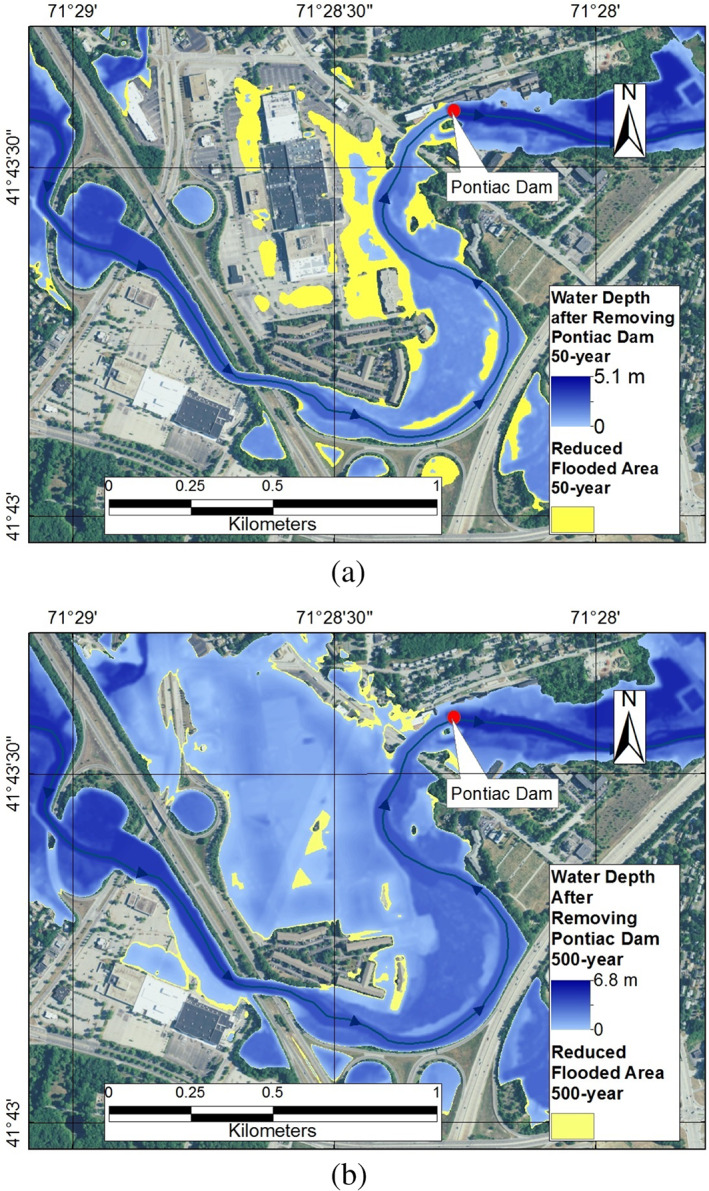
Impact of the Pontiac Diversion Dam on the flood. Reduced flooding areas (if the dam was removed) are shown in yellow: (a) 50‐year flooding event, and (b) 500‐year flooding event

The impact of Pontiac Dam on flooding was simulated in HEC‐RAS model for 50‐, 100‐, and 500‐year flooding events. Although many processes and variables such as flow speed, water depth, and sediment transport will be affected by dam removal (Bednarek, 2001), here, we only considered the inundation extent. Figure 15 shows the reduced inundation areas (if the dam is removed) corresponding to floods with various return periods. As this figure shows, the impact is noticeable for 50‐year event but not that significant for the larger 500‐year (or March 2010) event. In other words, the size of these diversion dams can be considered small compared to the huge impact of a very extreme flooding event. This is mainly because the main channel of a river accommodates a small portion of the flood discharge during large event (Bankfull discharge has usually a return period of about 2 years; Petit & Pauquet, 1997). Based on the results, it can be concluded that during the huge flood of March 2010 (which has a 500‐year return period), the existence of the Pontiac Dam did not significantly intensify the flood around the Warwick Mall.

Furthermore, Figure 4 indicates that an extreme flood event may lead to the failure of the dam. This failure may happen because these historical dams were not designed for the hydrodynamic loadings associated with the recent (such as March 2010 record‐breaking flood) and future extreme events; also, they have not been well maintained. Large volumes of contaminated sediments stored behind these dams would be released/redistributed upon failure or improper removal. These sediments may contain persistent high concentrations of contaminants deposited especially during the period before the Clean Water and Clean Air Acts in the 1970s (Corbin, 1989). As there are many historical dams in this river, further research is necessary to identify the contaminants in sediments (by coring) and simulate the possible contamination of the river as a result of dam‐break events. These studies should consider safe removal of dams that pose a significant contamination risk even if the flooding risk may not be reduced significantly.

As mentioned before, accumulation of debris upstream of bridges is another factor that can increase the flood risk. Here, the increased flood risk due to debris will be presented, as an example, for the Pawtuxet Village Bridge (Figure 5), which is located in a residential area. In the HEC‐RAS model, the height and the width of a block can be specified by the user (Brunner, 2001) to simulate debris. Based on previous guidelines (Lagasse, Clopper, Zevenbergen, Spitz, & Girard, 2010), the average width of the block should be considered up to 15 times of the pier width; the height of the block should be around 0.33–0.5 of the water depth. The pier width of the Pawtuxet Bridge is 2.2 m, the span of the river at the bridge is 28 m, and the water depth is about 1.5 m during a 100‐year flood. Therefore, a block of 20 m long and 0.5 m height was used to model the debris impact on flooding.

**FIGURE 16:**
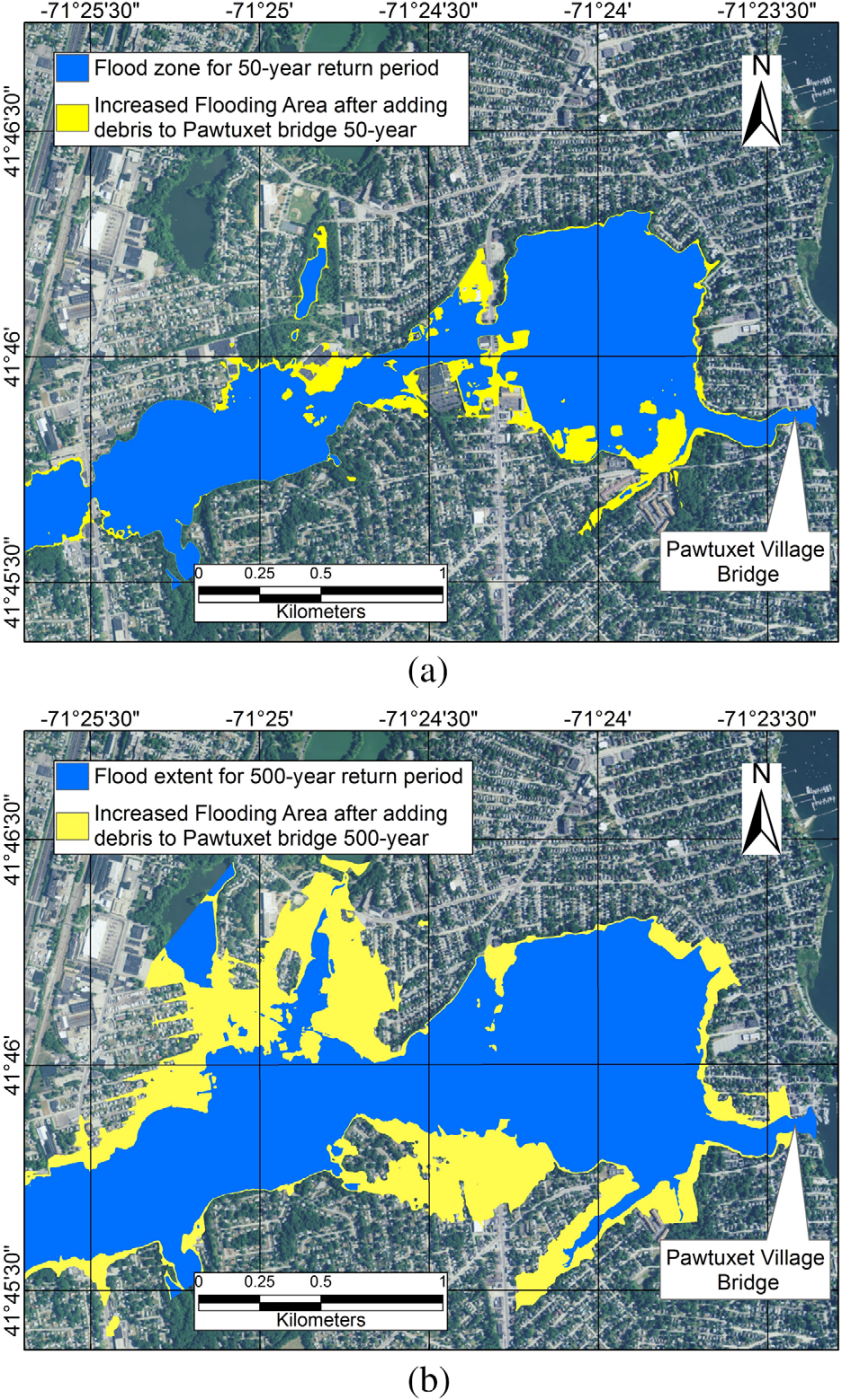
Change in flooding extent due to accumulation of debris at the Pawtuxet Village Bridge assuming (a) 50‐ and (b) 500‐year event scenarios

The impact of debris on the extent of flooding is shown in Figure 16. The inundated areas are compared before and after adding debris for 50‐ and 500‐year flooding events. In both events, there is a significant increase inundated area; contrary to dam removal case, the impact is higher for the larger return period (500 years). Also, the results shows a 2.1, 4, and 4.3 m of increase in water elevation due to debris accumulation for 50‐, 100‐, and 500‐year flooding events, respectively.

### 3.2 Flood risk in past and future

#### 3.2.1 Uncertainty in flood risk due to precipitation changes

**FIGURE 17:**
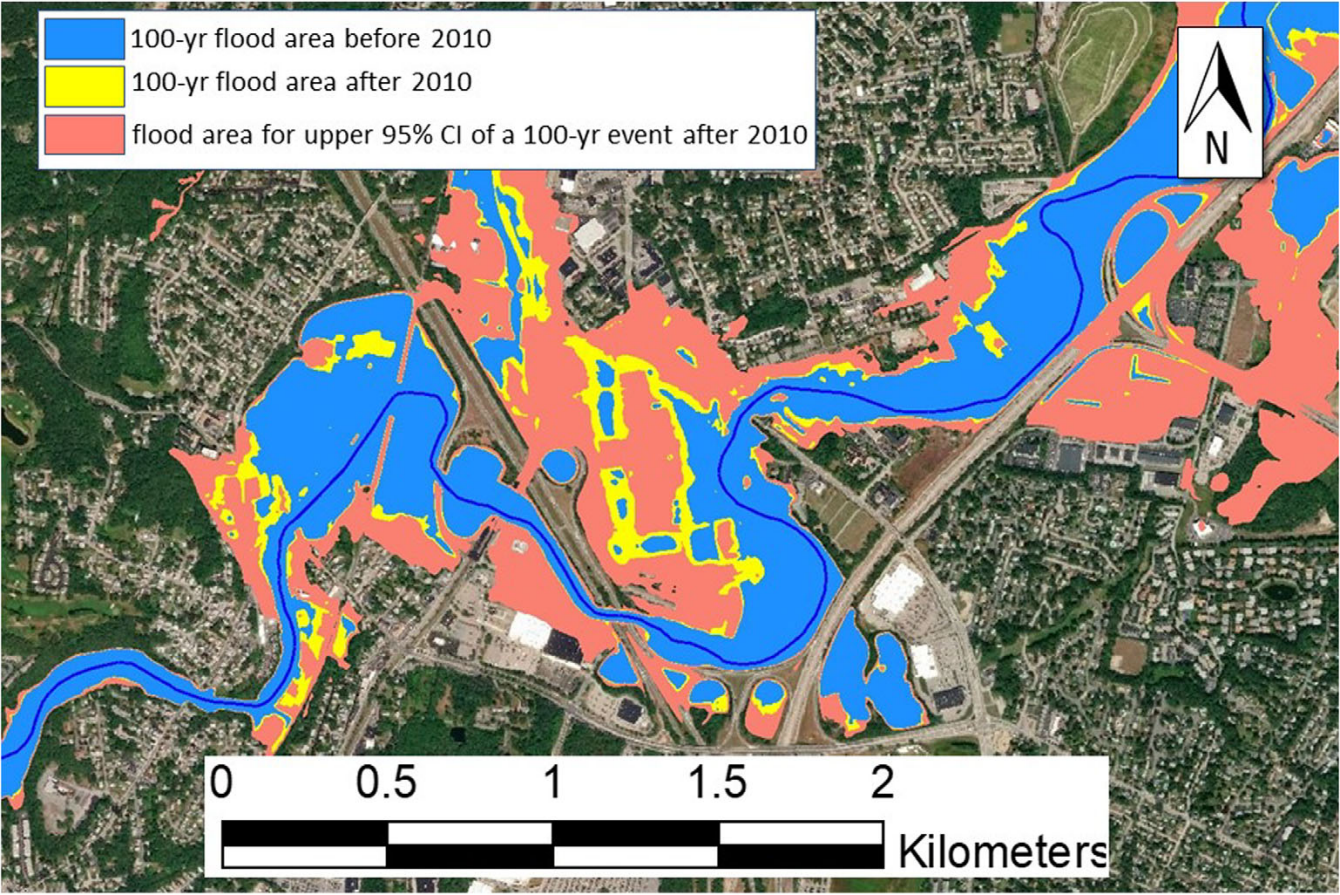
Change in the flood zone in the vicinity of the Warwick Mall due to the change in precipitation for a 100‐year event for before 2010, after 2010, and relative 95% confidence limits

Referring to Section 2.3, climate change has led to an increase in the precipitation rate in the study area. Here, we discuss how changes in extreme precipitation, and consequently in flood flow discharges, will result in a large uncertainty in the prediction of the 100‐year flood event which is a basis for determination of flood zones. The 100‐year flow discharge at the USGS 01116500 was 188 m^3^/s until 2009, and was was increased to 250 m^3^/s after the 2010 event (Zarriello et al., 2012). There is an additional uncertainty in the extreme value analysis as the peak flow data usually do not exactly follow a generalized extreme value distribution curve. This uncertainty is larger if only a few very extreme events (e.g., March 2010 event in RI) occur in a watershed. Therefore, confidence intervals are reported for the peak discharge values corresponding to various return periods. For the 100‐year event, the lower and upper 95% confidence limits are reported as 180 and 680 m^3^/s, respectively (Zarriello et al., 2012). The upper confidence limit is more than twice the mean value. Consequently, relying only on the mean predicted 100‐year event for flood risk assessments, given this large uncertainty is not justified. As Figure 17 shows many areas will be added to flood risk zone if instead of the mean 100‐year event, the upper confidence limit is used for risk assessment.

There is a significant uncertainty associated with mapping a flood zone or the area that can be inundated by a flood with a 100‐year return period. Therefore, there is a need to communicate this uncertainty to local communities living in these areas as well as flood plain managers. Several previous research have suggested methods to address the uncertainty in flood mapping; for instance, using flood probability maps (Di Baldassarre, Castellarin, Montanari, & Brath, 2009; Smemoe, Nelson, Zundel, & Miller, 2007), and using a probabilistic framework for flood mapping by employing coupled hydrologic and hydraulic models (Stephens & Bledsoe, 2020). This uncertainty has implications for flood insurance purposes since, currently, FEMA FIRM maps do not show the upper and lower confidence intervals of flood zones for a specific return period. Furthermore, it is necessary to reduce this uncertainty by adding/enhancing observational stations for precipitation and water level as suggested in this study. It is also necessary to continuously revise flood risk studies and numerical models that are the basis of flood risk maps, particularly after major hydrological events.

Therefore, in flood risk assessments and inundation mapping, the uncertainty due to trend in extreme precipitation and lack of sufficient data should be communicated to stakeholders and decision makers as suggested in previous research (Beven, Lamb, Leedal, & Hunter, 2015; Kuklicke & Demeritt, 2016).

#### 3.2.2 Sea level rise

**FIGURE 18:**
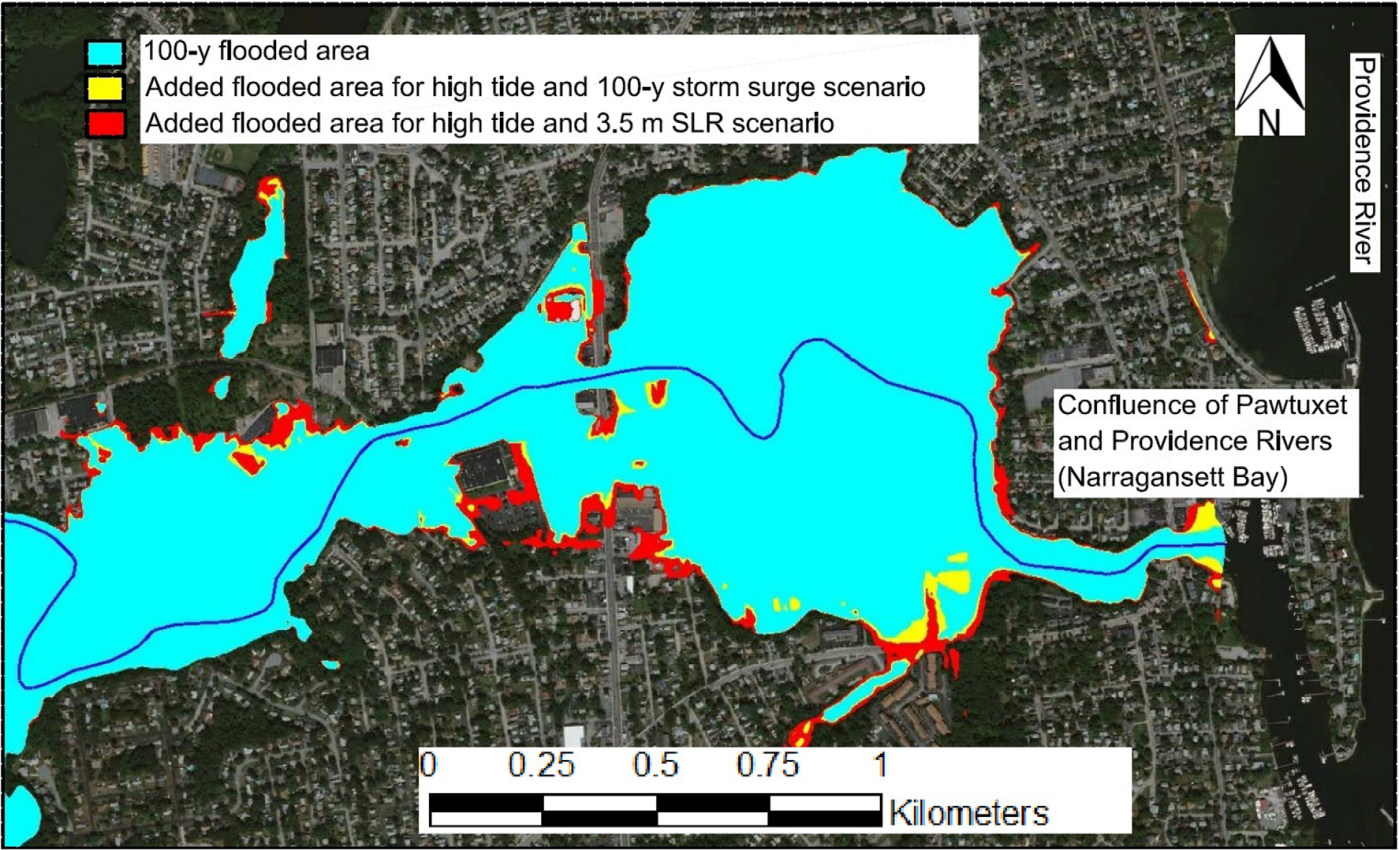
Comparison of the flooded area for a 100‐year flood event in the Main Branch assuming several scenarios at downstream of the river for tides (i.e., high tides), storm surge (100 year), and sea level rise (SLR; 3.5 m)

To assess the maximum impact of SLR on flooding extent, the flooded area for a 100‐year event was plotted assuming the current mean sea level, and was compared with the flood area for an extreme scenario; based on NOAA's recent estimation in this region (Grilli, Spaulding, Oakley, & Damon, 2017; Spaulding et al., 2020) if sea level rises 3.5 m (Figure 18). In both scenarios, it was assumed that flood occurs during high tide. As this figure shows, for this case study, the impact is not very significant compared with other risks (e.g., debris in the river). Nevertheless, impact of SLR on flooding is highly dependent on topography of the region, and may become very large for other regions (e.g., Wassmann, Hien, Hoanh, & Tuong, 2004). Figure 18 also compares the SLR scenario with combined inland and coastal flooding scenario (i.e., 100‐year inland and 100‐year storm surge) which shows a similar impact: a slight increase in the flooded area.

#### 3.2.3 Risk of wet coastal storms, Hurricane Rhody

To assess the potential impact of a plausible wet hurricane in future, a synthetic wet hurricane that was created in a recent study was considered (Stempel et al., 2018; Ullman et al., 2019). Hurricane Rhody is an extreme hypothetical or synthetic hurricane which was created based on the characteristics of the historical hurricanes that have severely impacted Northeastern United States. The tropical storm forms near the Bahamas and propagates northward on a similar track as of Hurricane Carol (1954). The forward speed of the storm is similar to that of 1938 New England Hurricane. The storm makes its first landfall as a strong Category 3 hurricane to the west of Rhode Island. After the initial landfall, the hurricane executes a loop, similar to Hurricane Esther (1961). It makes a second landfall in Rhode Island in which it is a weaker, slower Category 2 storm with a heavy rainfall. Although it is not possible to provide an accurate (or even an estimate) of the probability of this hurricane, several local and federal agencies^6^ have shown interest in using this storm for extreme risk assessments.

To estimate the rainfall produced by this synthetic hurricane, a simple parametric method was implemented (Lonfat, Rogers, Marchok, & Marks Jr, 2007; Tuleya, DeMaria, & Kuligowski, 2007). The Rainfall CLImatology and PERsistence (R‐CLIPER) model estimates the rainfall produced by the landfalling of tropical storms. R‐CLIPER is based on the satellite‐derived tropical cyclone rainfall observations, and has been used by National Hurricane Center to forecast the rainfall. It assumes a symmetric distribution of rainfall along the track of a tropical cyclone. The rainfall distribution depends on the storm intensity (maximum wind speed) and the storm size. This parametric model has been further refined (Tuleya et al., 2007) using rain gauge data and Tropical Rainfall Measuring Mission (TRMM) data (Kummerow, Barnes, Kozu, Shiue, & Simpson, 1998). Based on this model, the TRMM rainfall rate (or intensity in mm/hr or in/day) profiles and storm intensity can be correlated as follows (Tuleya et al., 2007):

(3) $$R\left(r,V\right)=\left\{\begin{array}{ll} R_{0}+\left(R_{m}-R_{0}\right)\frac{r}{r_{m}} & \;\text{for}\;r<r_{m},\\ R_{m}e^{-(\left(r-r_{m}\right)/r_{e})} & \;\text{for}\;r\;\geq \;r_{m}, \end{array}\right)$$

where *R* is the rain rate/intensity, *r* is the radius from the storm center, *V* is the tropical storm maximum wind which indicates its intensity. *R* varies linearly from *R*_0_ at *r* = 0 to maximum rain rate *R*_*m*_ at *r* = *r*_*m*_; it then decays exponentially for *r* ≥ *r*_*m*_. All parameters of this equation (i.e., *R*_0_, *R*_*m*_, *r*_*m*_, and *r*_*e*_) linearly depend on the maximum wind speed of a tropical storm; empirical linear regression equations have been provided for them based on observed data (Tuleya et al., 2007).

**FIGURE 19:**
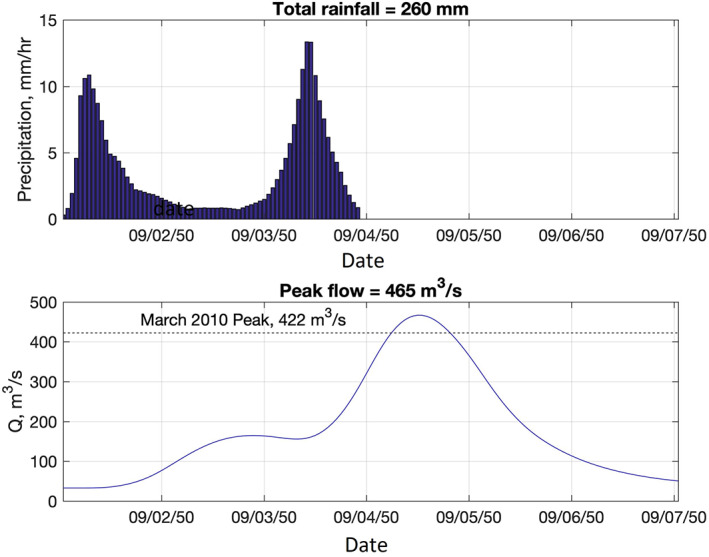
Time series of the rainfall generated by Hurricane Rhody in the Pawtuxet River Watershed (top), and the simulated discharge by HEC‐HMS at the USGS stream gauge in Cranston (bottom). The peak flow discharge of the record‐breaking March 2010 is shown for comparison. HEC‐HMS, Hydrologic Engineering Center's River Analysis System; USGS, United States Geological Survey

By implementing the R‐CLIPER model, the precipitation data were extracted for the Pawtuxet River Watershed during Hurricane Rhody. Since the size of the watershed is very small compared to the synthetic storm and its precipitation field, a uniform distribution of rain rate was assumed. As mentioned before, Hurricane Rhody has two landfalls on its track. Therefore, the precipitation and the storm surge generated by the Hurricane Rhody include two peaks during the storm. It is also assumed that the Rhody Hurricane occurs during 3 days (72 hr) from September 1, 2050 to September 4, 2050. Figure 19 shows the Hurricane Rhody's precipitation time series at the Pawtuxet River Watershed and the discharge calculated by HEC‐HMS model. The time series of discharge is calculated at the USGS stream‐gauge in Cranston, RI. The peak discharge for the Hurricane Rhody is 465 m^3^ which is slightly greater than the discharge for March 2010 event (i.e., 422 m^3^/s). It means that the return period for the flood caused by Hurricane Rhody at the Pawtuxet River is more than 500 years. Therefore, the risk of wet hurricanes should be considered in future risk assessments of flooding for both coastal and inland areas. The risk assessment can be conducted by building synthetic storms based on historical data. This will inform decision makers about possible combined inland and coastal flooding in coastal watersheds.

## 4 CONCLUSIONS

In this study, a detail assessment of several factors that can influence the flood risk was presented. This case study in the Pawtuxet Watershed resulted in the following conclusions that are important to consider in flood risk management policies and strategies, and are ignored in existing tools and databases such as FEMA FIRMs.

The management of river‐related structures such as reservoirs, diversion dams, and bridges can highly impact the flood risk zones. For instance, in this study, it was shown that regulating water level in the Scituate reservoir can decrease the risk of an extreme precipitation event (March 2010) from a 500‐year event to a 10‐year event downstream of the dam. In particular, new studies should be carried out to assess the feasibility of flood risk mitigation using existing reservoirs that currently operate only for water supply purposes. More advanced reservoir management techniques that consider uncertainty in flood risk and water supply maybe able to address both objectives of minimizing the risk and meeting the demand.

For this case study, it was shown that small diversion dams (e.g., historical textile mill dams) have more impact on the risk of more frequent floods while their impact on very extreme events is not that significant. Nevertheless, large volumes of contaminated sediments stored behind these dams (Corbin, 1989) would be released/redistributed upon failure after extreme floods or improper removal. These sediments may contain persistent high concentrations of contaminants deposited especially during the period before the Clean Water and Clean Air Acts in the 1970s. As there are many historical textile mill dams in this region, further research is necessary to identify the contaminants in sediments (by coring), and simulate the possible contamination risk as a result of dam‐break events.

It was shown that debris can highly increase the flood risk and consequently flood risk zones. Therefore, it is recommended to generate flood risk maps for similar regions for both scenarios (with and without debris). Otherwise, flood risk may be highly underestimated.

It was demonstrated that changes in extreme precipitation, and consequently in flood flow discharges, resulted in in a large uncertainty in the prediction of the 100‐year flood event and consequently flood zones in this area. In flood risk mapping, these uncertainties should be communicated to stakeholders and decision makers using methods that are presented in previous research.

Wet hurricanes can potentially pose a high risk of inland flooding. In particular, it was demonstrated that a synthetic hurricane with two landfalls (Hurricane Rhody) can generate a record‐breaking rainfall, while the first landfall of this hurricane did not lead to significant flood risk. It is recommended to predict the future flood risk associated with wet hurricanes by considering scenarios (i.e., synthetic hurricanes based on historical hurricanes) that can lead to more rainfall to better understand and assess this risk.

Changes in precipitation and predicted sea level rise, as other studies have shown, should be considered in flood risk management. It was shown that in this case study, extreme flooding has a trend, and historical data do not represent the future flood risk. Therefore, at least, an uncertainty should be included in flood maps that are generated based on the historical data. This uncertainty can be quantified based on trend in the historical data as well as climate model predictions.

In general, this study highlights several issues concerning the use of 100‐year maps for flood risk assessments. More comprehensive and probabilistic approaches that include the effects of river structures (dams, reservoir operation, and bridges/debris), possible changes in the future due to climate change (change in extreme precipitation and projected sea level rise), and other sources of uncertainty (e.g., land use changes) can provide a more reliable tool for flood risk management as well as communication of the risk to stakeholders and decision makers.

## Data Availability

I confirm that I have included a citation for available data in my references section.

## Endnotes

^1^
Property Claim Services: www.iii.org.

^2^

www.climatesolutionsne.org.

^3^

http://www.rigis.org/pages/2011-statewide-lidar-project-details.

^4^

http://www.rigis.org.

^5^

http://websoilsurvey.nrcs.usda.gov.

^6^
For example, U.S. Department of Homeland Security which has sponsored a project based on this hurricane.
